# Invasive pulmonary aspergillosis in patients with decompensated cirrhosis: case series

**DOI:** 10.1186/1471-230X-7-2

**Published:** 2007-01-31

**Authors:** Hélène Prodanovic, Christophe Cracco, Julien Massard, Camille Barrault, Dominique Thabut, Alexandre Duguet, Annick Datry, Jean-Philippe Derenne, Thierry Poynard, Thomas Similowski

**Affiliations:** 1 Assistance Publique – Hôpitaux de Paris, Service de Pneumologie et Réanimation, Groupe Hospitalier Pitié-Salpêtrière, Paris, France; 2 Assistance Publique – Hôpitaux de Paris, Service d'Hépato-Gastroentérologie, Groupe Hospitalier Pitié-Salpêtrière, Paris, France; 3 Assistance Publique – Hôpitaux de Paris, Service d'Hépato-Gastroentérologie, Hopital Henri-Mondor, Créteil, France; 4 Assistance Publique – Hôpitaux de Paris, Laboratoire de Parasitologie-Mycologie, Groupe Hospitalier Pitié-Salpêtrière, Paris, France

## Abstract

**Background:**

Opportunistic invasive fungal infections are increasingly frequent in intensive care patients. Their clinical spectrum goes beyond the patients with malignancies, and for example invasive pulmonary aspergillosis has recently been described in critically ill patients without such condition. Liver failure has been suspected to be a risk factor for aspergillosis.

**Case presentation:**

We describe three cases of adult respiratory distress syndrome with sepsis, shock and multiple organ failure in patients with severe liver failure among whom two had positive *Aspergillus *antigenemia and one had a positive *Aspergillus *serology. In all cases bronchoalveolar lavage fluid was positive for *Aspergillus fumigatus*. Outcome was fatal in all cases despite treatment with voriconazole and agressive symptomatic treatment.

**Conclusion:**

Invasive aspergillosis should be among rapidly raised hypothesis in cirrhotic patients developing acute respiratory symptoms and alveolar opacities.

## Background

Invasive pulmonary aspergillosis is a severe and often lethal infection. It mainly occurs in patients with malignancy who experience deep and prolonged neutropenia, but there are other established risk factors (e.g. high-dose long-term corticosteroid therapy or advanced forms of AIDS) [1]. Invasive pulmonary aspergillosis has recently been described in critically ill patients without malignancy [2-4], and Aspergilluss is increasingly considered as an emerging pathogen in non-hematological patients. Among the non-hematological patients diagnosed with invasive pulmonary aspergillosis in the critical care setting were patients with Child C liver cirrhosis [3]. This condition has thus be considered as a possible risk factor for invasive pulmonary aspergillosis, all the more so that acute liver failure is known to promote fungal infection [5]. In support of this contention and to raise the awareness of clinicians managing patients with decompensated cirrhosis, we report invasive pulmonary aspergillosis in three such patients.

## Case presentations

The three cases were diagnosed over a 18 months period, in two different hospitals.

### Case 1

A 54-year-old man was admitted for ascites and acute renal failure revealing Child-Pugh stage C13 alcoholic cirrhosis. A liver biopsy showed severe acute alcoholic hepatitis and treatment with prednisolone 1 mg/kg a day was started. Human immunodeficiency virus (HIV) serology was negative, as were viral hepatitis B (HBV) and viral hepatitis C (HCV) tests. Over the following 21 days, four episodes of hemorrhagic shock from grade II esophageal varices occurred. A transjugular intrahepatic portosystemic shunt was placed on day 21. On day 22, severe sepsis developed with middle lobe alveolar consolidation on the chest x-ray. Fiberoptic bronchoscopy with bronchoalveolar lavage (BAL) was performed before initiating empirical antibiotic therapy with piperacillin-tazobactam and ofloxacin. Microbiological examination of the BAL fluid evidenced the presence of mycelia, hence the addition of fluconazole to the treatment. On day 23, the patient deteriorated and was admitted to the ICU (Simplified Acute Physiology Score II -SAPS2-61, Organ dysfunction and/or Infection -ODIN 4/7). The chest x-ray radiograph showed bilateral diffuse alveolar opacities. The patient was mechanically ventilated (PaO2/FiO2158 mmHg). The total neutrophil count was 21,000/mm^3 ^(lymphocytes 219/mm^3^). Blood and urine samples, pleural and peritoneal fluids, and all catheters yielded negative cultures. Microbiological examination of repeated BAL fluid (performed on day 24) was negative, but the aspergillosis latex antigen agglutination test was positive at 1/256 (Platelia Aspergillus EIA for immunoenzymatic detection of galactomannan antigen of Aspergillus in serum; Bio-Rad, Marnes la Coquette, France). *Aspergillus *serology was negative (Protide Immunoelectrophoresis Aspergillus FSK1-MICROGEN, Beckman-Coulter, Fullerton, USA). Blood aspergillosis antigen tests (Platelia Aspergillus EIA for immunoenzymatic detection of galactomannan antigen of Aspergillus in serum; Bio-Rad, Marnes la Coquette, France) were positive on two occasions (possible false positive in the first instance because of the administration of piperacillin-tazobactam [6,7]). Fluconazole was switched to intravenous voriconazole (loading dose 400 mg two times on day 1, maintenance dose 200 mg twice daily) on day 27. The patient died on day 29. The family refused necropsy.

### Case 2

A 55-year-old man was first admitted for severe pneumonia with rhabdomyolysis, acute renal failure and encephalopathy, revealing Child-Pugh stage C12 alcoholic cirrhosis. Empirical antibiotic therapy with piperacillin-tazobactam and ofloxacin was started. The serology tests for HIV, HBV and HCV were negative. On day 2, septic shock developed and the patient was transferred to the ICU (SAPS2 92; ODIN 5/7). He was mechanically ventilated (PaO_2_/FiO_2 _104 mmHg), placed under continuous hemodiafiltration, and received hemodynamic support plus intravenous hydrocortisone hemisuccinate (50 mg every 6 hours, 6 days). Upon the results of initial blood cultures idenfying *Streptococcus pneumoniae *as the likely cause of the pneumonia, antibiotic therapy was switched to high dose amoxycillin (6 g daily). The patient improved. He could be weaned from mechanical ventilation after two weeks, and from continuous hemodiafiltration after three weeks. Mechanical ventilation was resumed two weeks later because of a catheter-related *Staphylococcus *infection causing septic shock. After one additional week, new bilateral pulmonary infiltrates appeared. Microbiological examination of BAL fluid was negative for bacteria, while the histopathological examination yielded signs of alveolar damage with alveolar hemosiderosis (Golde score 38). At this time, the total leucocyte count was 8,600/mm^3 ^(258 lymphocytes). BAL fluid and plugged telescopic catheter specimen grew *Aspergillus fumigatus *(five colonies). Two serum aspergillosis antigen tests were positive at a four days interval, both at 6.3 ng/ml (Platelia Aspergillus EIA for immunoenzymatic detection of galactomannan antigen of Aspergillus in serum; Bio-Rad, Marnes la Coquette, France). *Aspergillus *serology was negative (Protide Immunoelectrophoresis Aspergillus FSK1-MICROGEN, Beckman-Coulter, Fullerton, USA). Intravenous voriconazole was prescribed (loading dose 400 mg two times on day 1, maintenance dose 200 mg twice daily) but the patient developed multiple organ failure and died on the second day of this treatment. The family refused necropsy.

### Case 3

A 64-year-old woman with a C14 alcoholic cirrhosis (Child Pugh score) and a histologically proven alcoholic hepatitis was admitted after one month of oral corticosteroid therapy, with a diagnosis of hepatic encephalopathy, in the context of a *Klebsiella *urinary tract infection. Amoxicillin/clavulanic acid was prescribed. The serology tests for HIV, HBV and HCV were negative. Over the week following admission, the patient developed acute respiratory failure, with bilateral pulmonary infiltrates and severe hypoxemia (PaO2 57 mmHg with oxygen 10 L/min). There was fever (38.5°C) and a total leucocyte count of 10,100/mm^3 ^(300 lymphocytes). The patient was transferred to the ICU (SAPS2 39, ODIN 3/7) and fiberoptic bronchoscopy was performed. Microbiological examination of BAL fluid and bronchial aspiration showed hyphae, and both types of sample grew *Aspergillus fumigatus*. No other infectious agents were identified. Aspergillosis antigen tests were negative (Platelia Aspergillus EIA for immunoenzymatic detection of galactomannan antigen of Aspergillus in serum; Bio-Rad, Marnes la Coquette, France). The *Aspergillus *agglutination test was positive with one arch and a titer of *1/320 *(Protide Immunoelectrophoresis Aspergillus FSK1-MICROGEN, Beckman-Coulter, Fullerton, USA). Intravenous voriconazole was prescribed (loading dose 400 mg two times on day 1, maintenance dose 200 mg twice daily). The patient developed multiple organ failure with severe hepatic dysfunction and died 2 weeks after admission (third day of voriconazole treatment). The family refused necropsy.

## Conclusion

Although they did not meet the host-related criteria of the European Organisation for Research and Treatment of Cancer (EORTC), the first two of our cases fulfilled the definition of "probable" invasive pulmonary aspergillosis [1]. The third case fulfilled one host-related criteria (prolonged administration of systemic corticosteroids in the previous 60 days) and also qualified as "probable" on the EORTC grid [1]. Because there was no *Aspergillus *culture from a normally sterile site, no tissue biopsy and no necropsy, none of the cases can be classified as "proven". We however believe that these three patients who rapidly developed bilateral pulmonary infiltrates indeed suffered from invasive aspergillosis. Indeed, in the three cases, elements suggestive of the presence of *Aspergillus *were present concomittantly in respiratory samples and in the blood, whereas the non-mycologic microbiological workup was consistently negative. Of note, CT scans could not be performed, so we do not know whether the halo sign, considered of high diagnostic value in cancer patients, was present or not. The value of this sign is yet to be ascertained in critically ill patients. Similarly, our patients did not exhibit wheezes or hemoptysis, which are important clinical signs of invasive pulmonary aspergillosis.

Our patients presented several possible risk factors for invasive pulmonary aspergillosis. Steroid exposure was noted in the three cases, even though the EORTC host-related criteria were met in only one case. In one case, steroid exposure only consisted in the short duration stress dose of hydrocortisone that is currently routinely used in septic shock, but is has been shown shown that hydrocortisone promotes the growth of aspergillus in vitro [8]. In addition, it could be speculated that in critically ill patients, the threshold dose of corticosteroid that increases the risk of invasive aspergillosis is lower than in other patients because of the possible state of immunoparalysis associated with critical illness [9]. Corticosteroids are also known to promote aspergillosis in patients with underlying lung lesions [10]. These elements strengthen the notion that corticosteroids should be used with caution in critically ill patients. They suggest that in the presence of Aspergillus infection, stopping or tapering such treatments should at least be the matter of discussion. Of note, previous lung disease and lung structural damage due to pneumonia or acute respiratory distress syndrome were present in the three cases, and could have intrinsically promoted invasive aspergillosis [11]. Importantly, the three patients exhibited severe hepatic dysfunction. This condition deteriorates the host defense capacity. Cirrhosis is strongly associated with an increased risk of sepsis and acute respiratory failure [12]. Opportunistic infections usually associated with profound immunosuppression have been reported in cirrhotic patients otherwise free of immunosuppressive conditions or treatments [13]. T-lymphocyte count is inversely related to the severity of cirrhosis [14]. In addition, as mentioned in the introduction, acute liver failure promotes fungal infections [5]. Therefore, the occurrence of invasive pulmonary aspergillosis in patients with severe liver failure is not really surprising. It has been described in solid organ transplant recipients [15] including liver transplant recipients [16]. In the three patients described here, the overall leukocyte counts were normal but the the lymphocyte counts were very low at the time of diagnosis, and always dramatically lower than upon admission. We therefore consider these three patients as severely immunocompromised. This may contribute to explain why *Aspergillus *serology was negative in the first two patients and only weakly positive in the third one, a common finding in the presence of immunosuppression [17].

Finally, it must be noted that the fatal outcome in the three cases that we describe is not suprising in view of the very high mortality expected from the SAPS values at the outset and of the dismal prognosis of severe sepsis and multiple organ failure in patients with advanced liver failure.

This case series fuels the hypothesis that severe, decompensated liver cirrhosis may be considered as a host risk factor for the development of invasive aspergillosis [3]. However, this assumption should be put into perspective, since in all cases other risk factors for opportunistic infection were present, such as steroid exposure, antibiotic use and severe critical illness. In patients with advanced or decompensated liver cirrhosis and pneumonia, a primary or secondary fungal origin should be considered, if infiltrates progress in spite of adequate antibiotic therapy and respiratory insufficiency develops. Further diagnostic exploration is mandatory, including bronchoscopy and lavage, mycological sampling, and serology for Aspergillus antigen. Medical imaging may be contributive, but is less pathognomonic than in neutropenic patients. Early pre-emptive treatment with antifungals is warranted.

## Competing interests

The author(s) declare that they have no competing interests.

## Authors' contributions

HP, CC, JM, CB, DT and ADu all contributed to the collection of clinical data, literature review, and drafting the manuscript. ADa carried out the microbiological and serological diagnostic of aspergillosis. JPD and TP reviewed the patients' files critically, and reviewed the manuscript for intellectual content. TS supervised the whole process and provided major input to the manuscript in its final form. All the authors gave final approval of the submitted manuscript.

## Pre-publication history

The pre-publication history for this paper can be accessed here:


